# Elastic Fibres in Sputum

**Published:** 1909-02-27

**Authors:** 


### ELASTIC FIBRES IN SPUTUM.

The presence of elastic fibres in, or their absence from, sputum is a matter to which less attention is sometimes directed than should be the case. Tubercle bacilli, pus-cells, mucus, adventitious micro-organisms of various kinds may all abound in sputum without there being any advancing disease of the lung. The granulations covering the walls of phthisical vomicæ may give rise to sputum containing all the above, and yet from week to week the amount of lung tissue present remains the same. In such cases no elastic fibres are found in the sputum.

When lung tissue is being broken down by gradual extension of the tuberculous and ulcerative processes, on the other hand, there will be elastic fibres in the sputum in greater abundance or in less, according as much or little lung tissue is being destroyed each day. The only way of obtaining a definite proof of progressive lung destruction, and even some measure of the rate of that destruction, is by examination of the sputum for elastic fibres.

One of the simplest methods of such examination is as follows: The sputum is spread out in not too thin a layer upon perfectly clean microscope slides in the ordinary way, and allowed to dry spontaneously. When perfectly dry, but otherwise unfixed, the slides are put into Weigert's stain for elastic fibres for a quarter of an hour. After the staining, the films are washed and the excess of stain removed by immersion in a 1 per cent. solution of hydrochloric acid in alcohol. The preparations are then allowed to dry, after which they may be coated with cedar oil and examined under the microscope. The elastic fibres appear of a fine steel-blue colour, and they are readily to be distinguished from every other structure present.

